# A novel method for the management of proximal segment using computer assisted simulation surgery: correct condyle head positioning and better proximal segment placement

**DOI:** 10.1186/s40902-015-0023-3

**Published:** 2015-08-04

**Authors:** Yong-Chan Lee, Hong-Bum Sohn, Sung-Keun Kim, On-Yu Bae, Jang-Ha Lee

**Affiliations:** 1 Department of Oral and Maxillofacial Surgery, Bestian Seoul Hospital, 429, Dogok-ro, Gangnam-gu, Seoul 135-998 South Korea; 2 Department of Orthodontics, Eton Dental Hospital, Choonchun, Gangwondo 215-804 South Korea; 3 Department of Oral and Maxillofacial Surgery, College of Dentistry, Gangneung-Wonju National University, 7 Jukheon-Gil, Gangneung, Gangwondo 210-702 South Korea

## Abstract

Computer Assisted Simulation Surgery (CASS) is a reliable method that permits oral and maxillofacial surgeons to visualize the position of the maxilla and the mandible as observed in the patient. The purpose of this report was to introduce a newly developed strategy for proximal segment management according to Balanced Orthognathic Surgery (BOS) protocol which is a type of CASS, and to establish the clinical feasibility of the BOS protocol in the treatment of complex maxillo-facial deformities. The BOS protocol consists of the following 4 phases: 1) Planning and simulation phase, 2) Modeling phase, 3) Surgical phase, and 4) Evaluation phase. The surgical interventions in 80 consecutive patients were planned and executed by the BOS protocol. The BOS protocol ensures accuracy during surgery, thereby facilitating the completion of procedures without any complications. The BOS protocol may be a complete solution that enables an orthognatic surgeon to perform accurate surgery based on a surgical plan, making real outcomes as close to pre-planned outcomes as possible.

## Introduction

The establishment of a satisfactory surgical treatment objective (STO) and the performance of accurate surgery according to the STO are prerequisites for the best outcome of orthognathic surgery [1]. Traditionally, two-dimensional (2-D) STO has been the gold standard planning method for orthognathic surgery. The accuracy of 2-D STO is generally acceptable [2–4]. However, direct localization on the computer image has a higher accuracy and consistency than does conventional manual localization [5]. When comparing the linear measurements on lateral cephalograms to those obtained from cone-beam computed tomography (CBCT) scans, the values obtained in CBCT imaging are much closer to the actual distance [6]. Basically, traditional STO is based on 2-D images. The movement of the maxilla and the mandible in orthognathic surgery is performed in three-dimensional (3-D) space. Thus, 2-D STO may not accurately predict the 3-D movements of the jaws after orthognathic surgery. Each of these discrepancies may result in a less than ideal surgical outcome. In isolation, these discrepancies may be minor, but when added together, the results may be significant [7].

The goal of Computer Assisted Simulation Surgery (CASS) is to achieve better outcomes than those achieved with traditional methods. The authors have developed a new surgery protocol, known as the Balanced Orthognathic Surgery (BOS) protocol, which is a type of CASS. The BOS protocol comprises 4 phases: 1) Planning and simulation phase, 2) Modeling phase, 3) Surgical phase, and 4) Evaluation phase.

The authors found that the BOS protocol provides maxillofacial surgeons with useful tools to make the real outcome as close as possible to the planned outcome. Surgical interventions in 80 consecutive patients were planned and executed by the BOS protocol. The results were excellent, and the BOS protocol might be a complete solution to enable an orthognathic surgeon to perform accurate surgery based on a surgical plan. The detailed procedure is introduced and discussed in this study.

## The workflow of the bos protocol

1. The planning and simulation phase

The transformation that occurs in orthognathic surgery is affine transformation, which exhibits translation, as well as rotation. The authors define the direction of jaw movement in the 3-D coordinate system as follows:

① The X-axis is the axis that depicts the direction from left to right.
② The Y-axis is the axis that depicts the direction from inferior to superior.
③ The Z-axis is the axis that depicts the direction from anterior to posterior.

**Fig. 1:**
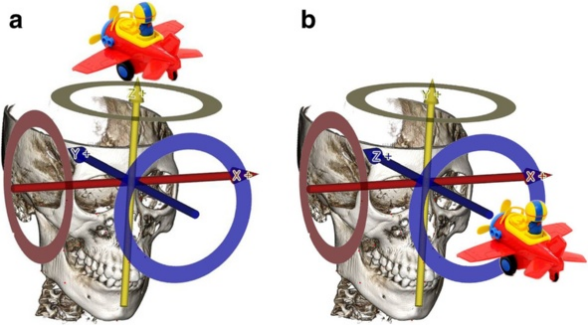
Three dimensional coordinate system according to the operator’s point of view. **a**. The view above the object’s head. **b**. The view in front of the object’s head

The authors’ 3-D coordinate system is defined from the operator’s point of view, which means that the Y-axis and Z-axis can be mutually exchanged compared with a currently used 3-D coordinate system in the currently used 3-D programs. The currently used 3-D coordinate system was developed on the assumption that the observer looks at the object from above the object’s head (Fig. 1a).

However, the anatomical location is defined based on the assumption that observer looks at the object face-to-face. Therefore, health care providers, such as doctors or dentists, are used to interpreting the 3-D coordinate system from a medical or dental perspective. For this reason, the authors defined the X, Y, Z axis based on the accustomed anatomic locations, such as Fig. 1b.

**Fig. 2:**
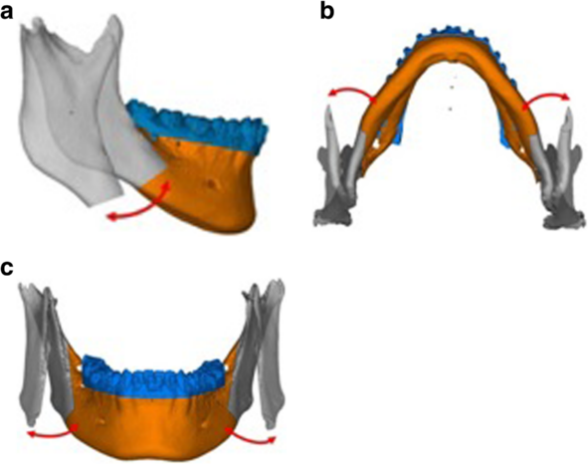
Proximal segment rotation in each plane. **a**. Proximal segment-sagittal rotation (P-SR). **b**. Proximal segment-axial rotation (P-AR). **c**. Proximal segment-coronal rotation (P-CR)

The authors used the following anatomic terms to depict the movement of both the mandible and maxilla in whole and/or in part. The term sagittal rotation was used to represent rotation or pitching, coronal rotation was used to represent canting or rolling, and axial rotation was used to represent yawing (Fig. 2).

**Fig. 3:**
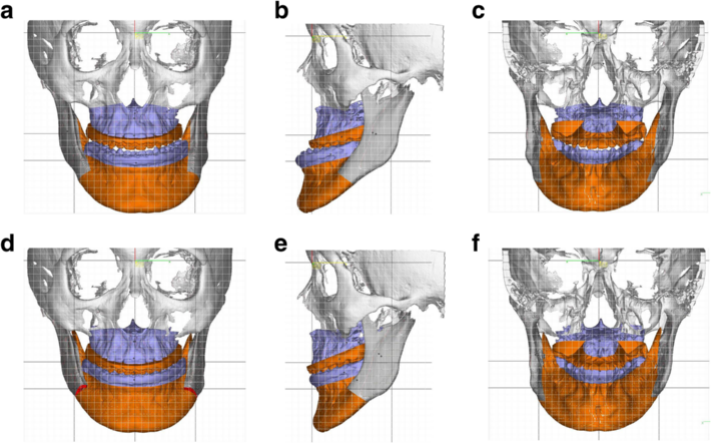
Comparing with preoperative image and three dimensional (3-D) surgical treatment objective (STO) image. **a**. Preoperative image (*frontal view*). **b**. Preoperative image (*lateral view*). **c**. Preoperative image (*rear view*). **d**. 3-D STO image (*frontal view*). **e**. 3-D STO image (*lateral view*). **f**. 3-D STO image (*rear view*)

**Fig. 4:**
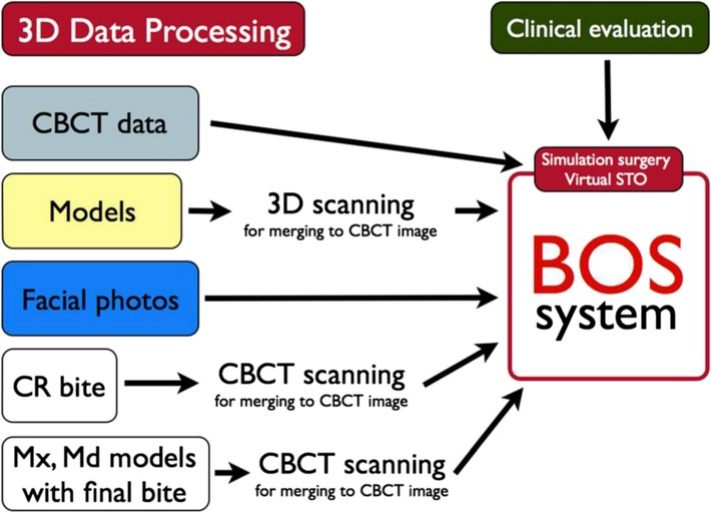
Planning and simulation phase

**Fig. 5:**
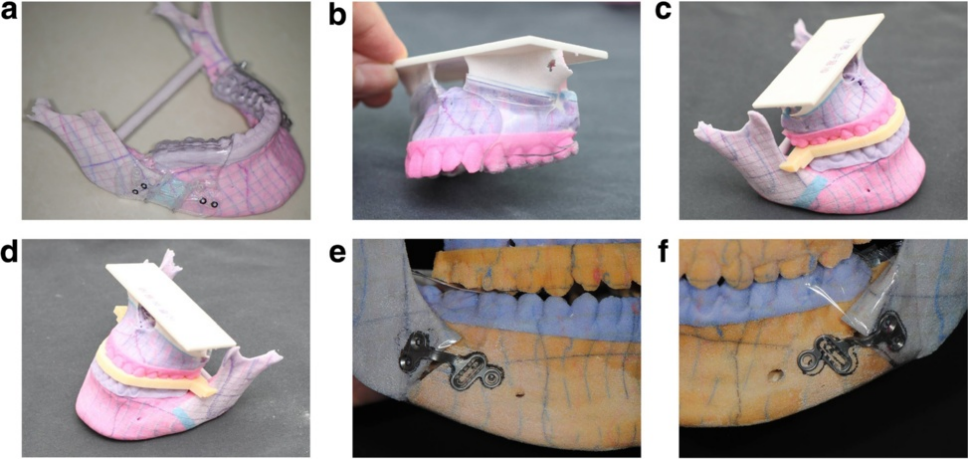
Modeling phase. **a**. Cutting guide (*mandible*). **b**. Cutting guide (*maxilla*). **c**. Computer-generated surgical stent (*right side*). **d**. Computer-generated surgical stent (*left side*). **e**. Pre-bent plates along the post-operative RP-model surface (*right side*). **f**. Pre-bent plates along the post-operative RP-model surface (*left side*)

**Fig. 6:**
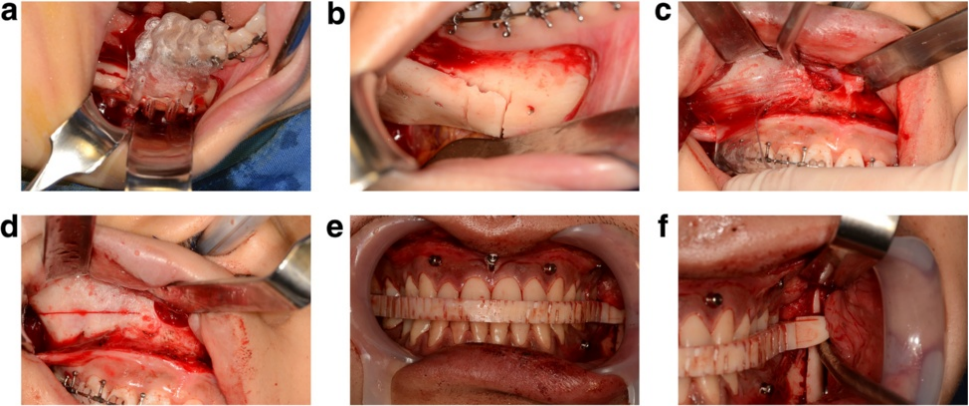
Surgical phase. **a**. Mounting a cutting guide on mandible. **b**. After osteotomy with cutting guide. **c**. Mounting a cutting guide on maxilla. **d**. After osteotomy with cutting guide. **e**. Mounting a computer-generated surgical stent (*anterior view*). **f**. Mounting a computer-generated surgical stent (posterior view)

**Fig. 7:**
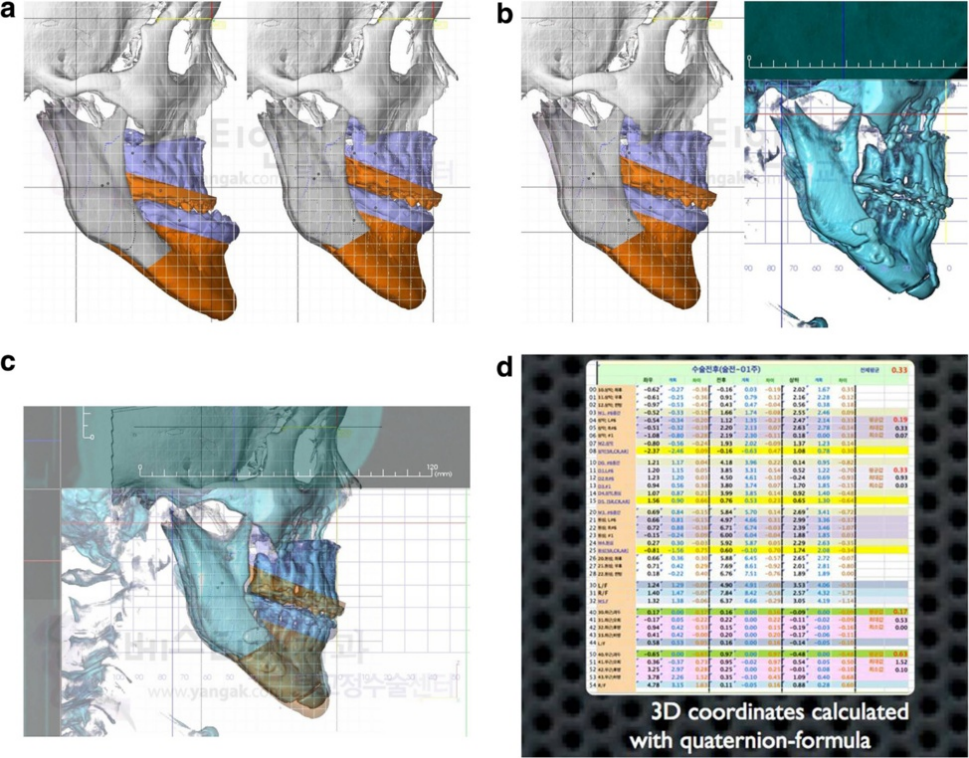
Evaluation phase. **a**. Simulated surgical image (*Left: Pre-operative view, Right: Post-operative view*). **b**. Post-operative simulated surgical image (*left*) and post-operative computerized tomogram (CT) image (*right*). **c**. Superimposition with postoperative simulated surgical image and postoperative CT image. **d**. Three dimensional coordinates calculated with quaternion-formula

① Proximal Segment-Sagittal Rotation (P-SR): The degree value of the rotation of the proximal segment rotated on the sagittal plane (degree).
② Proximal Segment-Axial Rotation (P-AR): The degree value of the rotation of the proximal segment rotated on the axial plane (degree).
③ Proximal Segment-Coronal Rotation (P-CR): The degree value of the rotation of the proximal segment rotated on the coronal plane (degree).

In the planning and simulation phase, a computerized composite skull model of the patient is generated to accurately represent the skeleton and the dentition by merging the scanned image of the dentition with the CT image of the skull. In addition, by establishing reference points, the patient’s neutral head posture (NHP) is recorded and transferred to the 3-D models. The authors can simulate orthognathic surgery according to our BOS equation (Fig. 3).

2) The modeling phase

In the modeling phase, surgical wafers, which also function as condyle positioning devices, are generated in the computer and fabricated by a rapid prototyping machine. Surgical cutting guides are made using a preoperative 3D RP model to perform the accurate cutting of the mandible and maxilla, and miniplates are pre-bent along the external surface of the postoperative 3D RP model to place the bony segments as close as possible to the planned position (Figs. 4 and 5).

3) The surgical phase
  In the surgical phase, the maxillofacial surgeon performs orthognathic surgery using the aforementioned surgical tools (Fig. 6).
4) The evaluation phase
  In the evaluation phase, post-OP CT images are superimposed on the simulated surgical image, and the differences between them are evaluated (Fig. 7).

## Clinical report

**Fig. 8:**
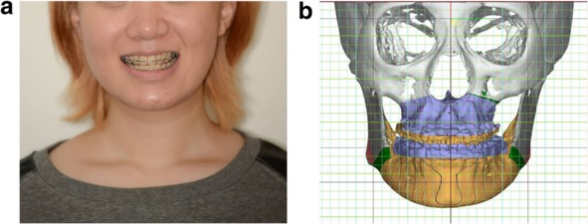
Preoperative application of the Balanced Orthognathic Surgery (BOS) protocol. **a**. Preoperative facial image. **b**. Preoperative simulated surgical image

**Fig. 9:**
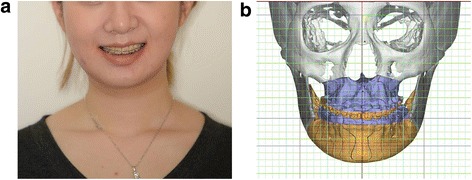
Postoperative results of the Balanced Orthognathic Surgery (BOS) protocol. **a**. Postoperative facial image. **b**. Postoperative simulated surgical image

A 20-year-old female patient was transferred from her local orthodontic clinic to the department of Oral and Maxillofacial Surgery of the Bestian Seoul Hospital in December 2013 to treat her protruded mandible and retruded maxilla. Her treatment followed the BOS protocol and was completed without any side effects and complaints (Figs. 8 and 9).

**Fig. 10:**
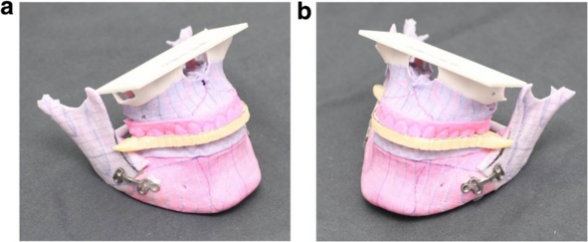
Computer-generated intermediate wafer and pre-bent plates act as splints to precisely reposition the proximal segments. **a**. Image of right side. **b**. Image of left side

## Review

Bilateral sagittal split ramus osteotomy (BSSRO) is the most widely used procedure to treat jaw deformities [8]. Although BSSRO is considered to be one of the most stable orthognathic procedures, there are still drawbacks to the BSSRO procedure. One of the major drawbacks of BSSRO is short-term and long-term post-surgical relapse. There are many factors that contribute to surgical relapse. The method of fixation, the amount of advancement and set back movement, the distraction of the condyle, the displacement of the proximal segment, idiopathic condylar resorption, and the interaction of the para-mandibular connective tissues are considered to be the possible causes of relapse [9–18]. Although the exact mechanism of relapse is not fully understood, many previous studies have demonstrated that it is multi-factorial in nature [19–21].

Schendel and Epker found that poor surgical techniques are related to the relapse of surgery [22]. They showed that post surgical distraction of the condyle and the poor positioning of the proximal segment and improper skeletal fixation are the major findings in surgical relapse. Many of the encountered problems were related to surgical technique. They commented that postsurgical distraction of the condyle was consistently associated with relapse. They also found that the positioning of the proximal segment and adequate skeletal fixation were keys to preventing relapse. Becktor et al. [10] suggested that control of the proximal segment is the most important aspect in preventing relapse. The general concern is that if the proximal segment becomes dislocated, it may predispose the patient to relapse [11]. Thus, great care is taken during surgery to ensure that the proximal segment and the condyle are not displaced from the glenoid fossa.

Post-surgical condylar resorption is thought to be one of the main causes of relapse of bilateral sagittal split ramus osteotomy. Many authors have noted that condylar resorption occurs if the condyles are placed too far superiorly and posteriorly [12, 13]. Ellis and Hinton showed that animals in which there was evidence of posterior displacement of the condyles showed evidence of resorption of the posterior surface of the condyle and anterior surface of the post-glenoid spine [12]. Arnett used the special term “condylar sag” to depict the displacement of the proximal segment in various forms [14]. He defined condylar sag as the inferior or anterior-inferior positioning of the condyle away from the seated position in the glenoid fossa. If condylar sag occurs, it has been shown that the condyle will return to its preoperative position within 8 weeks of surgery. This is observed as skeletal relapse. The anterior-inferior displacement of the condyle and increased posterior facial heights were considered to be the most important factors that contribute to relapse [15].

Long-term dental and skeletal stability after surgery is necessary for the precise positioning of the condyle [16–18]. Every surgeon pays special attention to positioning the proximal segment as close as possible to the planned position. Recently, condyle positioning devices have been developed to help surgeons to return the proximal segment to its original position. Rotskoff et al. [22] showed that using a condyle positioning device is useful in improving the repositioning of the proximal segment in a vertical and horizontal position, but there was no improvement in preventing the rotation of the proximal segment.

The authors also believe that the poor management of the proximal segment is the main cause of both short-term and long-term surgical relapse. In this newly developed BOS protocol, the authors used prebent plates and intermediate and final wafers that function in condyle positioning to act as splints to precisely reposition the proximal segments (Fig. 10). Hsu SS et al. showed that using CASS, the computerized plan may be consistently and accurately transferred to the patient to position the maxilla and the mandible at the time of surgery [23]. Baker SB et al. evaluated the accuracy of outcomes in computer-assisted simulation surgery in orthognathic surgery and showed that the CASS system proved to be an effective mechanism to treatment plan cases and prepare surgical splints for patients undergoing othognathic surgery [24].

In a study that compared the efficiency of bi-maxillary orthognathic surgery using CASS with cases planned with traditional methods, Schwartz noted that an average of 60 min were saved in planning each bi-maxillary surgery [25]. Our clinical experiences suggest that the maxillofacial surgeon’s goal in orthognathic surgery, such as achieving the correct condyle head position, maintaining the planned frontal ramal inclination, effectively placing the proximal and distal segments, and restoring mandibular symmetry, can be achieved using the BOS protocol. The use of 3-D soft tissue simulation in bi-maxillary surgery is accurate for clinical use [26]. Virtual planning for orthognathic surgery appears to be an accurate and reproducible method [27, 28]. Virtual surgical planning also improves surgical outcomes in obstructive sleep apnea patients [29]. Compared to the conventional method, 3-D surgical planning eliminates the need for dental impressions and simplifies the necessary technical steps [30].

However, it is important to note the drawbacks of the BOS protocol. To facilitate the use of the protocol, an intermediary technician is required. The RP model and wafers should be fabricated by an outside laboratory. Like other 3-D surgical planning methods [31], an increased cost of production is involved. When these drawbacks are mitigated, centers that treat orthognathic patients will replace traditional STO and model surgery with the BOS protocol.

## Conclusion

The BOS protocol might be a complete solution to enable an orthognatic surgeon to perform accurate surgery based on a surgical plan, making real outcomes as close as possible to pre-planned outcomes. When applying this protocol to corrective surgery for facial asymmetry, it can produce better results than those achieved using traditional methods.
